# Experimental and Analytical Evaluation of Strengthening Techniques for Ultra-High-Performance Concrete Circular Columns

**DOI:** 10.3390/polym18202541

**Published:** 2026-10-16

**Authors:** Aref Abadel, Hussein Elsanadedy, Husain Abbas, Mohammed Al-Mahbashi, Tarek Almusallam, Yousef Al-Salloum

**Affiliations:** Chair of Research and Studies in Strengthening and Rehabilitation of Structures, Department of Civil Engineering, College of Engineering, King Saud University, P.O. Box 800, Riyadh 12372, Saudi Arabia; aabadel@ksu.edu.sa (A.A.); helsanadedy@ksu.edu.sa (H.E.); habbas@ksu.edu.sa (H.A.); malmhbashi@ksu.edu.sa (M.A.-M.); musallam@ksu.edu.sa (T.A.)

**Keywords:** UHPC columns, strengthening, CFRP, steel jacketing, NSM, WWM

## Abstract

This study presents an experimental investigation to evaluate the effectiveness of four schemes for strengthening circular reinforced concrete (RC) columns. The columns were cast using ultra-high-performance concrete (UHPC). Ten RC columns, categorized into five groups including four strengthening schemes, were tested under concentric axial compression. The first group (G1) included the unstrengthened specimens (control specimens). Group G2 included specimens strengthened using two carbon fiber-reinforced polymer (CFRP) layers and 4Ø12 near-surface-mounted (NSM) steel rebars. Group G3 consisted of specimens strengthened using three CFRP layers. In group G4, a 3 mm thick steel tube was used for column jacketing. The specimens of group G5 were strengthened using UHPC jacketing reinforced with three welded wire mesh (WWM) layers and 4Ø8 longitudinal steel rebars. The fibers of CFRP layers in all schemes were oriented in the circumferential direction. The experimental results indicated that all strengthening schemes significantly increased ultimate load by 30% to 69% and initial stiffness by up to 130%. The G5 strengthening scheme (UHPC jacketing reinforced with WWM and 4Ø8 steel rebars) was most effective in enhancing load capacity and stiffness, while CFRP wrapping (G3) offered a superior balance of strength and ductility without increasing column dimensions, preserving post-peak integrity.

## 1. Introduction

Strengthening deficient reinforced concrete (RC) structures offers a viable method for enhancing structural performance, thereby ensuring compliance with contemporary design codes. This strengthening strategy presents a more sustainable alternative to the complete demolition and subsequent reconstruction of RC structures. Consequently, this approach is beneficial for resource conservation, encompassing time, financial expenditure, and material usage, while also contributing to a reduction in the overall carbon footprint linked to the construction sector [1]. The imperative for strengthening interventions may arise from a multitude of causes, encompassing structural aging, concrete degradation, modifications in building utilization and load-bearing demands, design deficiencies, reinforcement corrosion, and inaccuracies encountered during the construction phase.

In the past three decades, researchers have focused on developing effective methods for strengthening RC columns [2]. These methods aim to improve structural performance by increasing strength, ductility, and drift, while also considering other important factors. These factors include minimizing the costs and difficulties of implementation, reducing interference with normal building occupancy during the work, preserving the structure’s aesthetics, maintaining or improving long-term durability, and maintaining safe working conditions. The initial strengthening methods, such as steel jacketing and concrete encasement, improved the structure’s ability by increasing the size of the columns. Therefore, researchers have been developing and studying techniques that require fewer changes to the RC member geometry while also increasing its strength [3,4,5,6,7,8].

Ultra-high-performance concrete (UHPC) is an emerging and advanced cementitious material known for its excellent mechanical characteristics [9,10,11]. However, the brittleness of the UHPC mixture is a significant disadvantage; thus, adding steel fibers to the UHPC mixture can help reduce this problem. This mixture is characterized by its high strength, improved fracture toughness, ability to absorb energy, ductility, and better performance in the post-cracking phase [12,13,14].

Fiber-reinforced polymers (FRPs) are now recognized as a compelling substitute for traditional strengthening methods. A lot of research has been conducted worldwide to understand different aspects of using FRPs for strengthening [6,7,8]. Recently, hybrid jacketing has become prominent because it effectively combines the benefits of various strengthening methods and materials. As a result, this approach has become the main focus of recent research. Carbon fiber-reinforced polymer (CFRP) materials have been the predominant choice for the retrofitting of concrete columns over the past three decades among all FRP composites. Most of the research employed wrapping of CFRP sheets to reinforce RC columns [15]. This strengthening approach involves enveloping RC columns with multiple CFRP layers, which are distinguished by their exceptional characteristics, including elevated tensile strength, a lightweight composition, and resistance to corrosion [16]. Applying CFRP sheets to columns serves as external reinforcement, providing confinement to the column’s concrete and improving its flexural capacity and ductility [17]. The efficacy of this method relies on the bond between the CFRP and the column’s concrete, which facilitates efficient load transfer and stress redistribution. This alteration augments the load-bearing capacity and improves the column’s resilience against various loading scenarios, such as seismic loads [18]. Additionally, CFRP sheets can offer benefits including easy implementation, reduced added self-weight of the RC column, and suitability for a range of column geometries and dimensions [17,19].

Strengthening strategies encompass various approaches, such as section modification, steel tubes, near-surface mounted (NSM) rebars, and welded wire mesh (WWM) jacketing [20,21,22,23,24]. Each of these methods offers distinct benefits and drawbacks, which are contingent upon considerations of labor intensity, long-term durability, resistance to corrosion, fire performance, and overall cost-effectiveness [25]. The WWM method employs several WWM layers, which are fabricated from steel and arranged around the perimeter of the RC column. This mesh creates an effective, grid-like structure that conforms to the column’s surface [26,27]. The bond is critical for the effective transfer of loads at their interface. Improving this bond subsequently enhances the efficacy of the WWM jacket [28]. Consequently, the WWM application enables the bolstering of the column’s structural integrity by facilitating uniform load distribution, thereby reducing stress concentrations and augmenting resistance to lateral forces. By providing confinement to the concrete core, this process strengthens its load-bearing capacity and ductility [29,30]. Although WWM is relatively simple to use and often less expensive, it may not be as effective as other methods, such as CFRP wrapping or UHPC jacketing [29,31]. Moreover, WWM may corrode over time, so it is important to carefully consider environmental conditions and the need for long-term maintenance [32].

The strengthening of the RC column using steel jacketing (SJ) is a significant component in the realm of composite construction due to its integration of the beneficial properties of the constituent materials. In circular concrete-filled steel tube (CFST) columns, the confinement allows the concrete to be subjected to a triaxial state of stress, resulting in enhanced compressive performance relative to an unconfined column [33,34]. The presence of the steel tube facilitates the employment of high-strength concrete by providing confinement that mitigates its brittle and potentially explosive failure [3,35]. The integration of these characteristics results in an efficient structural member suitable for critical loading conditions, enhancing stiffness, strength, and ductility [36,37]. The investigations [38,39,40,41] focused on the enhancement of columns through a hybrid system that incorporates NSM reinforcement rebars alongside externally bonded GFRP (glass FRP) or CFRP sheets. The findings indicated an enhancement in load capacity and stiffness when subjected to axial compression, which was linked to the role of NSM rebars in enhancing flexural resistance.

Recent studies have explored advanced FRP-based and hybrid strengthening systems for concrete columns. Zeng et al. [42] investigated FRP spiral-strip confinement and demonstrated improvements in the strength and deformation capacity of concrete. Hybrid systems combining FRP with UHPC or UHP-ECC have also shown considerable potential for enhancing the axial capacity and deformation behavior of circular and square concrete columns [43,44]. These studies demonstrate the potential of combining confinement and high-performance cementitious materials for effective column strengthening.

Although CFRP confinement, NSM reinforcement, steel jacketing, and concrete/UHPC jacketing have individually been investigated as strengthening techniques, their effectiveness has predominantly been evaluated in separate studies involving different column geometries, concrete strengths, reinforcement details, and loading conditions. In particular, published evidence remains limited regarding the strengthening of existing UHPC columns, and a systematic comparison of fundamentally different strengthening mechanisms under identical experimental conditions is lacking. Consequently, their relative effects on the strength, stiffness, ductility, energy absorption, and failure mechanisms of UHPC columns remain insufficiently understood. To address this gap, the present study provides a controlled experimental comparison of four strengthening strategies representing different strengthening mechanisms: CFRP confinement combined with NSM longitudinal reinforcement, CFRP confinement alone, steel jacketing, and reinforced UHPC jacketing. The objective is not merely to rank the techniques according to strength enhancement but to establish how the different strengthening mechanisms influence axial capacity, stiffness, failure behavior, and deformation and energy-absorption characteristics, and to assess the ability of simplified analytical approaches to predict their axial capacities. Ten circular RC columns of UHPC were tested under concentric axial compression and categorized into five distinct groups. The first group (G1) included the unstrengthened specimens (control specimens). The G2 group included strengthening the specimens using 4Ø12 NSM steel bars with wrapping of two CFRP sheets. The G3 group consisted of strengthening specimens using three CFRP layers. The G4 group employed steel jacketing with a 3 mm steel tube for strengthening of specimens. The specimens of the G5 group were strengthened using UHPC jacketing reinforced with three WWM layers and 4Ø8 longitudinal steel bars.

## 2. Materials and Methods

### 2.1. Raw Materials

#### 2.1.1. Concrete Mixture (UHPC)

The UHPC mix used was a specialized blend consisting of cement, micro silica, crushed sand, steel fibers, superplasticizers, and water with their proportions of 900, 222, 1000, 78.5, 30, and 164 kg/m^3^, respectively (for additional details on the mix preparation, see Refs. [45,46]). Thus, the water-to-binder ratio was about 0.15. The compressive and splitting tensile strengths of UHPC, determined using cylindrical specimens measuring 100 mm × 200 mm, were 137 MPa and 18 MPa, respectively. The test columns were prepared and tested as per ASTM C1856 [47]. The specimens were water-cured for 28 days prior to testing. In this study, steel fibers with a hooked-ended shape, 30 mm long, and 0.55 mm in diameter were employed at a content of 1% by volume. The tensile strength and elastic modulus of these fibers were 1345 MPa and 200 GPa, respectively.

#### 2.1.2. Steel Rebars

In this study, deformed steel rebars were employed. The longitudinal rebars had a diameter of 10 mm, while ties had a diameter of 8 mm in all columns. Additionally, 12 mm diameter bars were used in one of the strengthening schemes. The yield stresses for the 12, 10, and 8 mm diameter rebars were 585, 512, and 480 MPa, respectively. The ultimate stresses, determined following ASTM A370 [48], were 665, 564, and 510 MPa, respectively, while the elastic modulus of all bar sizes was 200 GPa.

#### 2.1.3. WWM

For column strengthening, WWM was utilized. The WWM of 0.9 mm wire diameter had a 13 mm square grid. The modulus of elasticity of the wires was 106 GPa, whereas the yield and ultimate stresses were 382 and 544 MPa, respectively.

#### 2.1.4. CFRP

Unidirectional CFRP sheets, each 0.6 mm thick, were used in this study. Standard coupon testing was used to determine the CFRP sheets’ tensile strength in accordance with ASTM D3039 [49]. Table 1 lists the properties of the CFRP laminate and the epoxy resin used.

#### 2.1.5. GFRP

In groups G1, G4, and G5, the column ends were strengthened with 3 layers of a commercially available GFRP/epoxy composite system having a layer thickness of 1.3 mm. The tensile strength of the GFRP sheets was obtained through coupon testing following ASTM D3039 [49]. Table 1 lists the GFRP laminate’s characteristics.

#### 2.1.6. Steel Pipe

A steel pipe was used to strengthen the columns. The thickness of the selected steel pipe was 3 mm. The pipe had yield and ultimate stresses of 382 and 544 MPa, respectively, and an elastic modulus of 200 GPa, determined in accordance with ASTM-E8 [50].

### 2.2. Specimen Preparation

**Figure 1:**
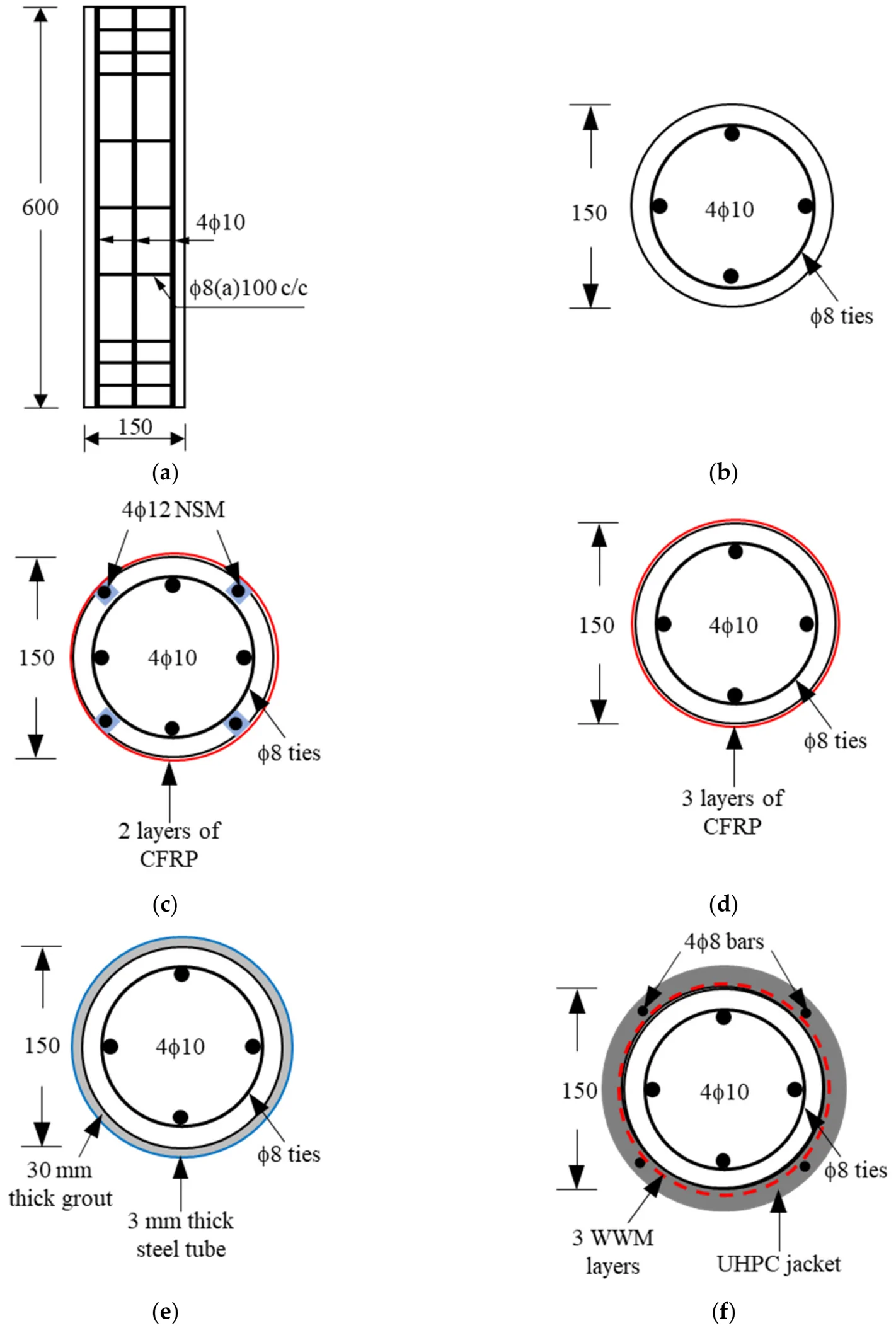
Reinforcement detailing of different columns: (**a**) Longitudinal section of control column; and cross-sections of column groups: (**b**) G1; (**c**) G2; (**d**) G3; (**e**) G4; and (**f**) G5 (Unit: mm).

This study involved testing RC circular columns of 150 mm diameter and 600 mm height. Four steel bars of 10 mm diameter were used in all columns as longitudinal reinforcement (giving a steel ratio of 1.78%), while 8 mm bars at 100 mm spacing were used for the transverse reinforcement (ties). To reduce the probability of premature failure at the specimen ends and achieve optimal load-carrying capacity, the tie spacing at the column ends was decreased to 50 mm, and GFRP strips were also used at the column ends. All columns were provided with a 20 mm concrete cover. The RC columns in this study have been classified into five groups (Table 2). The first group (G1) was used as the control specimens, which were constructed without strengthening. The second group (G2) included the specimens strengthened using 4Ø12 NSM steel rebars and two CFRP layers. The third group (G3) consisted of the specimens strengthened using three CFRP layers. In the fourth group (G4), the steel jacketing with a 3 mm thick steel pipe was used for strengthening. The specimens in the fifth group (G5) were strengthened using UHPC jacketing reinforced with three WWM layers and 4Ø8 longitudinal steel rebars. Each column configuration was tested in replicate; therefore, a total of ten columns were tested. The strengthening schemes were proportioned to target broadly comparable improvements in axial load-carrying capacity based on the applicable design/analytical approaches. However, identical strength enhancement was neither imposed nor expected because the investigated techniques rely on different materials, confinement mechanisms, and load-transfer mechanisms. Figure 1 shows the reinforcement details of the columns.

**Figure 2:**
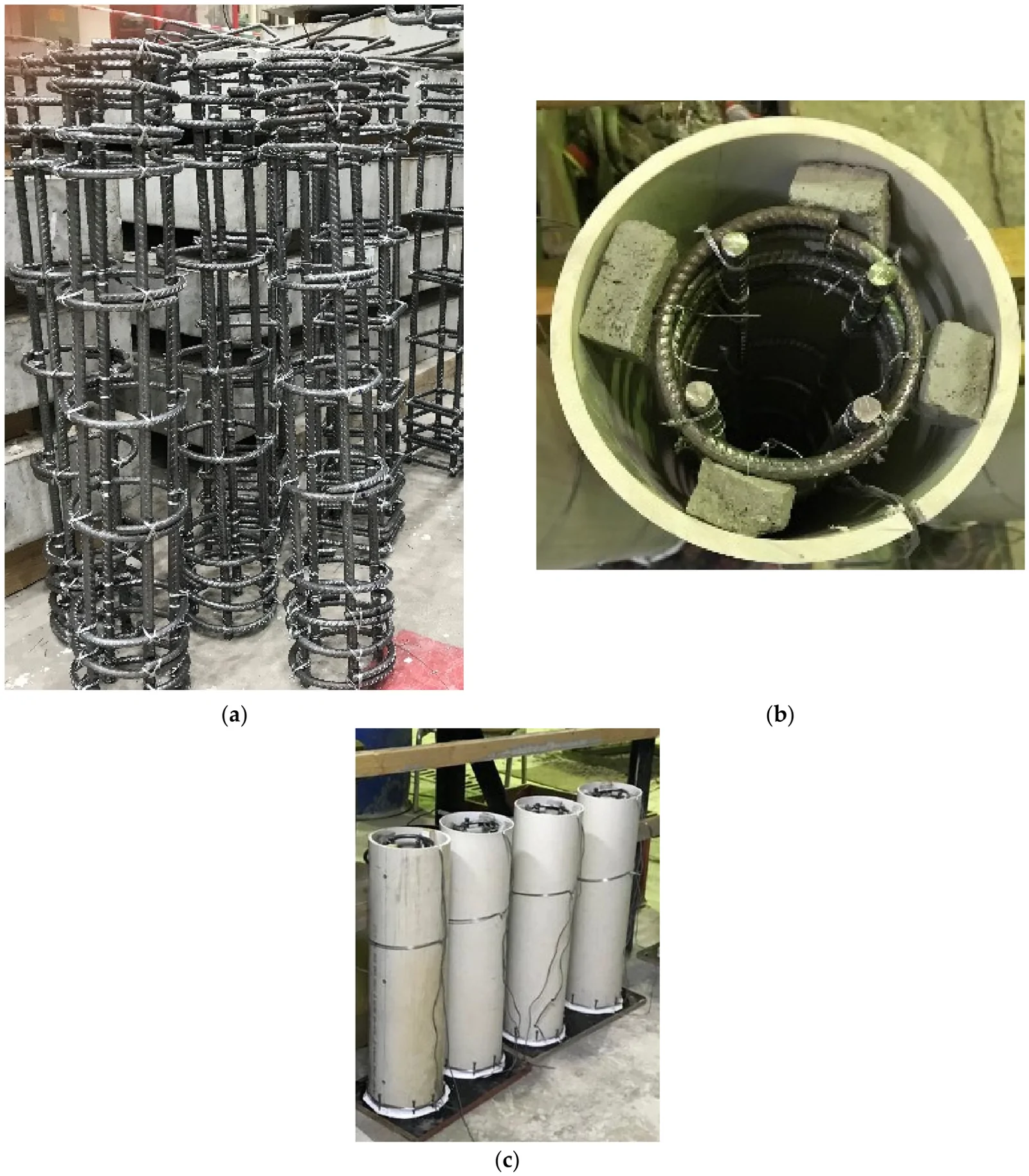
The specimen preparation: (**a**) Rebar cages; (**b**) Rebar cage placed inside PVC pipe; and (**c**) Specimens ready for casting.

The details of column preparation are illustrated in Figure 2. A vibrating table was utilized during concreting to reduce the air trapped in the concrete. After 24 h of casting, the specimens were demolded by removing the PVC pipe molds and subsequently water-cured for 28 days.

### 2.3. Column Strengthening Techniques

This study employed four strengthening strategies, all of which aimed to produce columns of comparable strength. The column ends were strengthened with FRP sheets to avoid premature failure of the columns.

#### 2.3.1. NSM and CFRP Confinement

**Figure 3:**
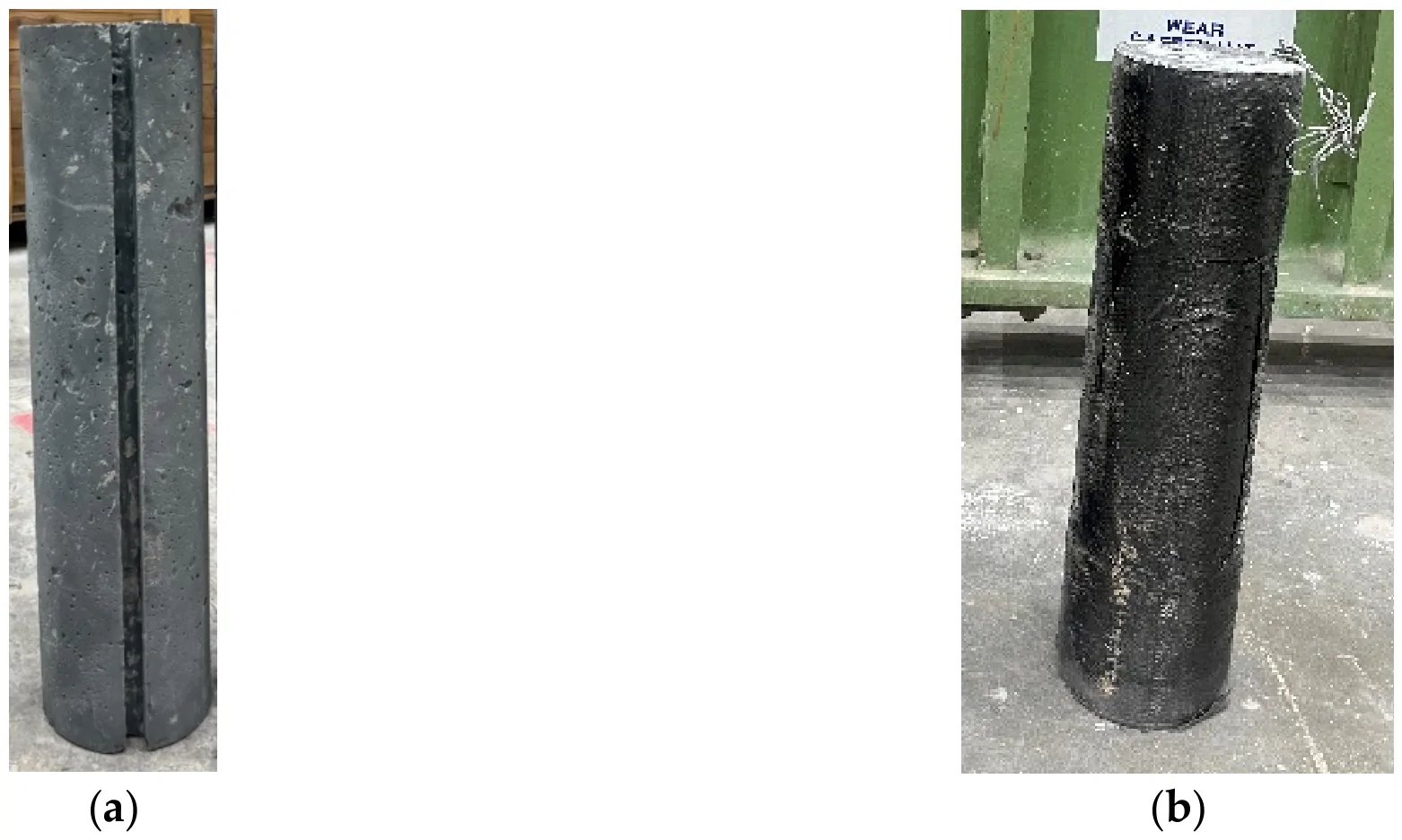
Strengthening of G2 specimens: (**a**) Specimens showing NSM grooves; and (**b**) Specimens after strengthening.

The strengthening technique for G2 specimens consisted of a set of longitudinal NSM steel rebars (4Ø12) and the wrapping of two CFRP layers with fibers oriented in the circumferential direction. Figure 3 illustrates the strengthening of the G2 specimens. The vertical grooves for the NSM rebars were created using 20 mm square wooden pieces, which were placed within the specimen mold, as shown in Figure 3a. After the curing of the columns, the NSM rebars were positioned within the grooves, which were subsequently filled with epoxy adhesive Sikadur 31 (tensile strength = 15 MPa and elastic modulus = 4300 MPa). Before the wrapping of CFRP sheets using the wet-layup procedure, sandblasting was used to roughen the surface and remove any contaminants, thereby enhancing the bond strength between CFRP sheets and the concrete. The process of CFRP wrapping involved the application of a thin epoxy resin layer to the surface of the specimen. Then, the first epoxy-impregnated CFRP layer was applied over the specimen using a roller to eliminate any entrapped air. The application procedure was repeated to install the second CFRP layer (Figure 3b). The CFRP sheet was overlapped by 100 mm. To avoid the direct load application on the CFRP sheets, a 5 mm unwrapped gap was maintained at both ends of the specimens. It should be noted that an additional CFRP layer was added at the column ends to avoid premature local failure.

#### 2.3.2. CFRP Confinement

The G3 specimens were strengthened using three layers of CFRP sheets, with the fibers oriented in the hoop (i.e., circumferential) direction to provide confinement. Surface preparation was carried out to ensure adequate bonding, including cleaning and sandblasting of the concrete surface. The CFRP layers were then applied following the same wet layup procedure described above. Similar to group G2 specimens, an additional CFRP layer was added at the column ends to prevent local premature failure.

#### 2.3.3. Steel Jacketing

**Figure 4:**
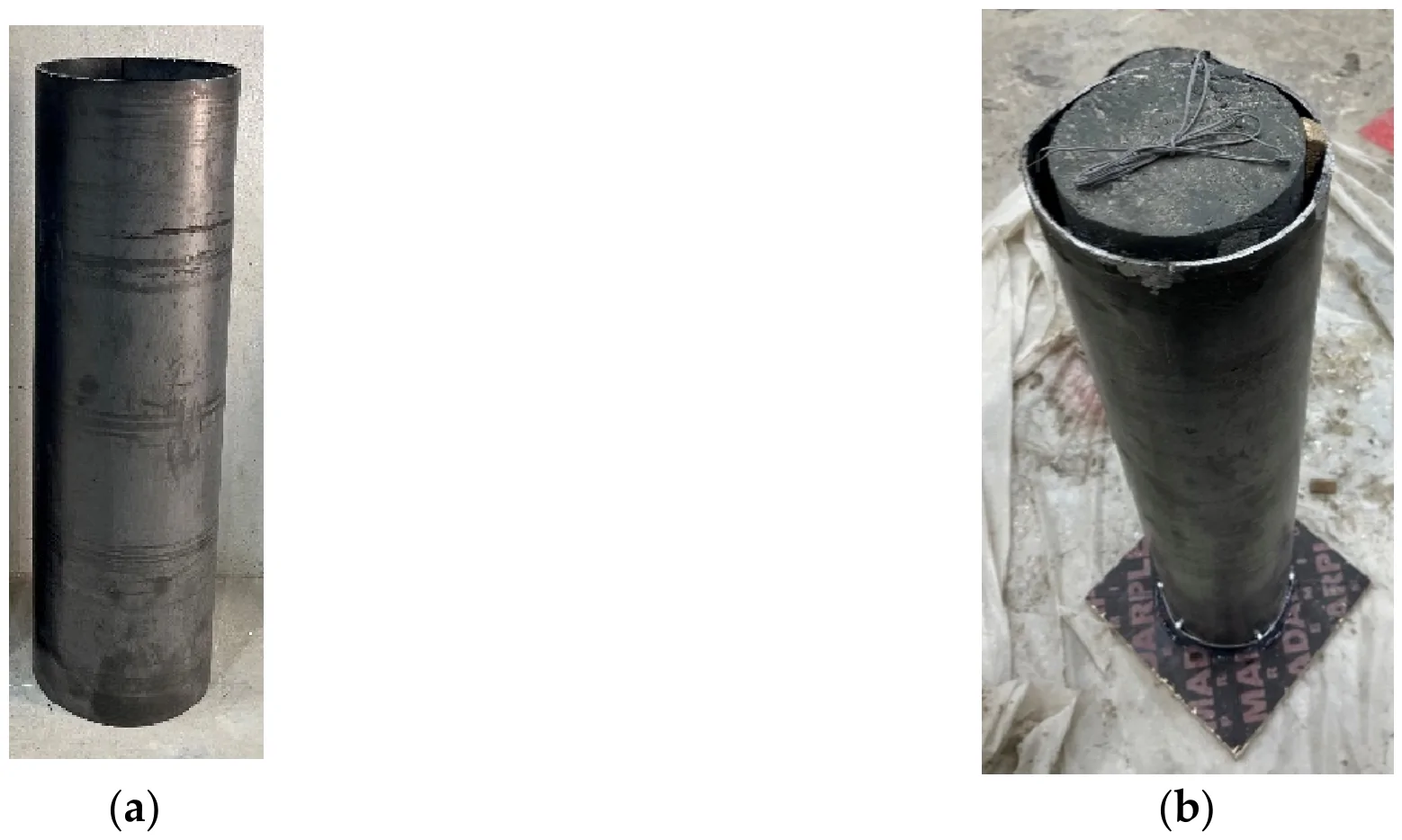
The G4 specimens’ strengthening: (**a**) Steel tube; (**b**) G4 specimen before grout casting.

The G4 specimens were strengthened using a steel jacketing system consisting of a 3 mm-thick steel tube and grout concrete infill. Prior to strengthening, the column surface was cleaned to remove dirt and contaminants, and then sandblasted to achieve adequate surface roughness. The surface was subsequently cleared of residual dust. Figure 4 illustrates the G4 specimens’ strengthening. The steel tube was cut to the right size based on the height of the specimens. To avoid direct loading of the steel tube, it was kept 3 mm short in height. The concrete column was cast independently and subsequently positioned within the steel tube, as illustrated in Figure 4b. After that, Sikadur 42 MP was poured into the gap between the column and the steel pipe, providing a 30 mm thick grout layer around the column. The practical field application of this scheme will be different. In situ, this method would require pre-fabricating the jacket in two halves by bending steel plates, which should then be positioned around the existing column and welded. Finally, the filling of the annular space between the steel jacket and RC column using a high-strength grout to ensure proper composite action will be the same.

#### 2.3.4. UHPC Jacketing Reinforced with WWM and Longitudinal Rebars

**Figure 5:**
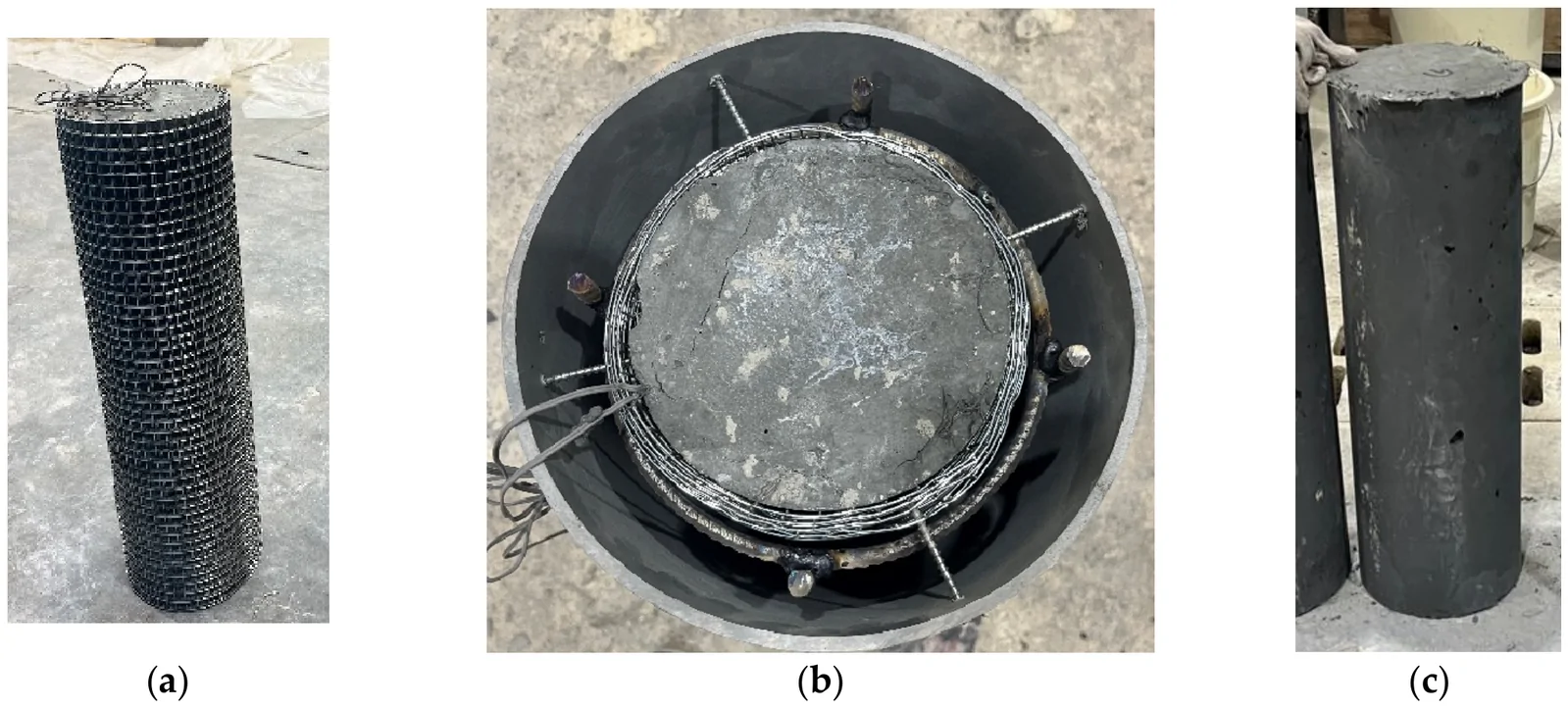
The G5 specimens’ strengthening: (**a**) application of WWM layers; (**b**) column with WWM layers and steel rebars inside mold; and (**c**) specimens after strengthening.

The G5 specimens were strengthened using UHPC jacketing reinforced with three layers of WWM and four Ø8 longitudinal steel rebars. Before strengthening, the specimen surface was cleaned to remove contaminants, sandblasted to enhance surface roughness, and subsequently cleared of residual dust. Figure 5 shows the strengthening procedure for the G5 specimens. The WWM layers were affixed to the specimen’s surface and attached using binding wires to ensure a tight fit, as shown in Figure 5a. A 100 mm overlap was maintained in the WWM layers. In addition, 4Ø8 longitudinal rebars were provided (Figure 5b). Finally, the UHPC was poured into the mold to form a uniform jacket with an overall thickness of 30 mm (Figure 5c). The concrete cover for the added rebars was 10 mm.

### 2.4. Testing Method

**Figure 6:**
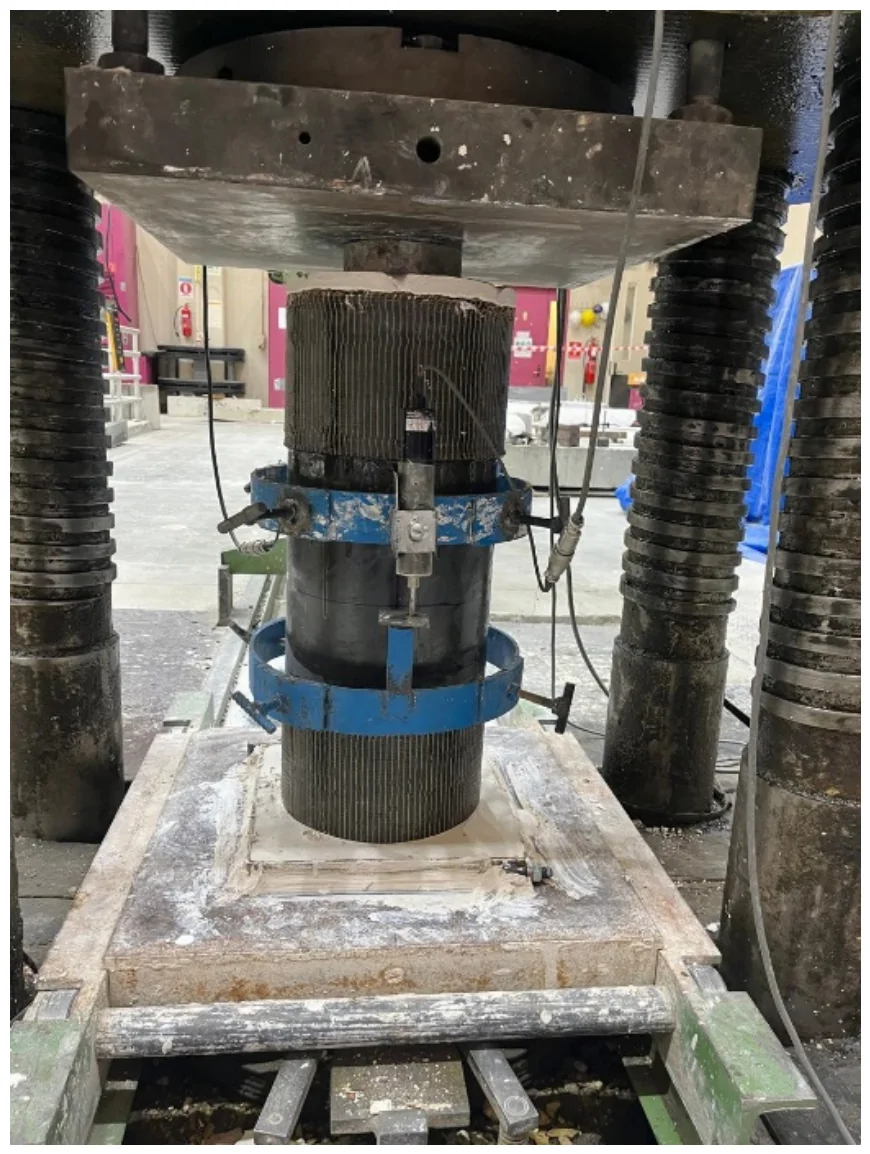
Test setup.

The specimens were experimentally tested using a universal testing machine (UTM) of 10,000 kN capacity (Alfred J. Amsler & Co., Schaffhausen, Switzerland). To monitor axial deformation, a compressometer equipped with two LVDTs (gauge length of 200 mm) was employed and mounted at mid-height of the columns, as depicted in Figure 6. Strains in the specimens were measured using electrical resistance strain gauges. Two strain gauges were mounted on the longitudinal steel rebars, and two additional gauges were installed on the concrete surface to record transverse and axial strains. In the CFRP-strengthened columns, two horizontal strain gauges were positioned at the mid-height of the CFRP jacket to record its hoop strain. Gypsum was applied to the column ends for proper alignment and uniform load distribution during testing. All specimens were loaded to failure at a constant displacement rate of 0.5 mm/min.

## 3. Results and Discussion

### 3.1. Failure Modes

**Figure 7:**
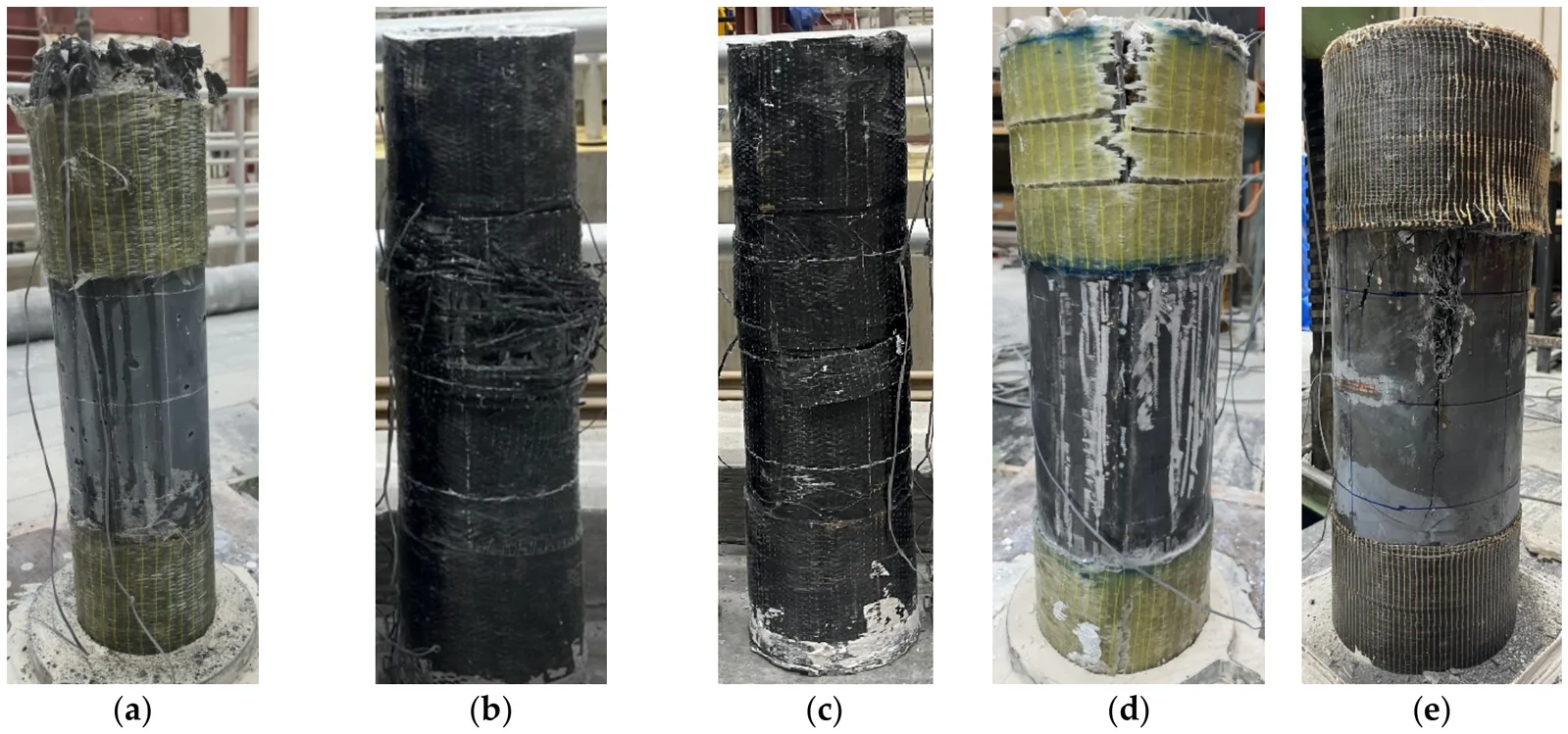
Failure patterns of the tested columns: (**a**) U-C; (**b**) U-CFRP-NSM; (**c**) U-CFRP; (**d**) U-SJ; and (**e**) U-WU.

Figure 7 depicts the failure patterns of all tested specimens. The failure patterns show how different strengthening systems behave and how they interact with the RC column under axial compression. In general, the anticipated failure mode in unconfined columns under compression involves concrete crushing accompanied by compression yielding of longitudinal steel bars. However, adopted strengthening techniques significantly altered both the failure progression and the final mode of collapse. The control column (U-C) failed by concrete crushing and a sudden brittle collapse, as shown in Figure 7a. The strengthened G2 column, retrofitted using NSM bars and CFRP wrapping (U-CFRP-NSM), failed at the middle third and displayed significant degradation and rupture of the CFRP sheet (Figure 7b). The occurrence of ringed rupture of the CFRP sheet can be ascribed to the tensile rupture of the CFRP sheet along the concrete perimeter, as illustrated in Figure 7b. The presence of NSM grooves in these specimens may have locally disturbed the otherwise uniform confinement mechanism; however, this effect is a possible contributing factor rather than a confirmed failure mechanism because local CFRP strains and interface stresses were not measured.

The strengthened G3 column, retrofitted by wrapping CFRP sheet (U-CFRP), showed almost the same failure pattern as was observed in the U-CFRP-NSM column. However, the failure was less violent in comparison to the G2 columns, indicating a more gradual loss of confinement through ringed rupture of the CFRP sheet (Figure 7c). Utilizing steel jacketing in the strengthening of the G4 column resulted in the core concrete expansion under compression, which exerted outward pressure on the steel wall, causing the fracture at the weld seam of the steel tube (Figure 7d). Figure 7e depicts the failure patterns noted in the G5 specimen (U-WU), strengthened with a UHPC jacket reinforced with WWM and longitudinal steel rebars. As the load increased, minor surface cracks in the UHPC mortar began to develop and grow until failure. The U-WU specimen’s failure was caused by the rupture of WWM and the cracking of the UHPC jackets near the mid-height of the specimen.

### 3.2. Load–Displacement Response

**Figure 8:**
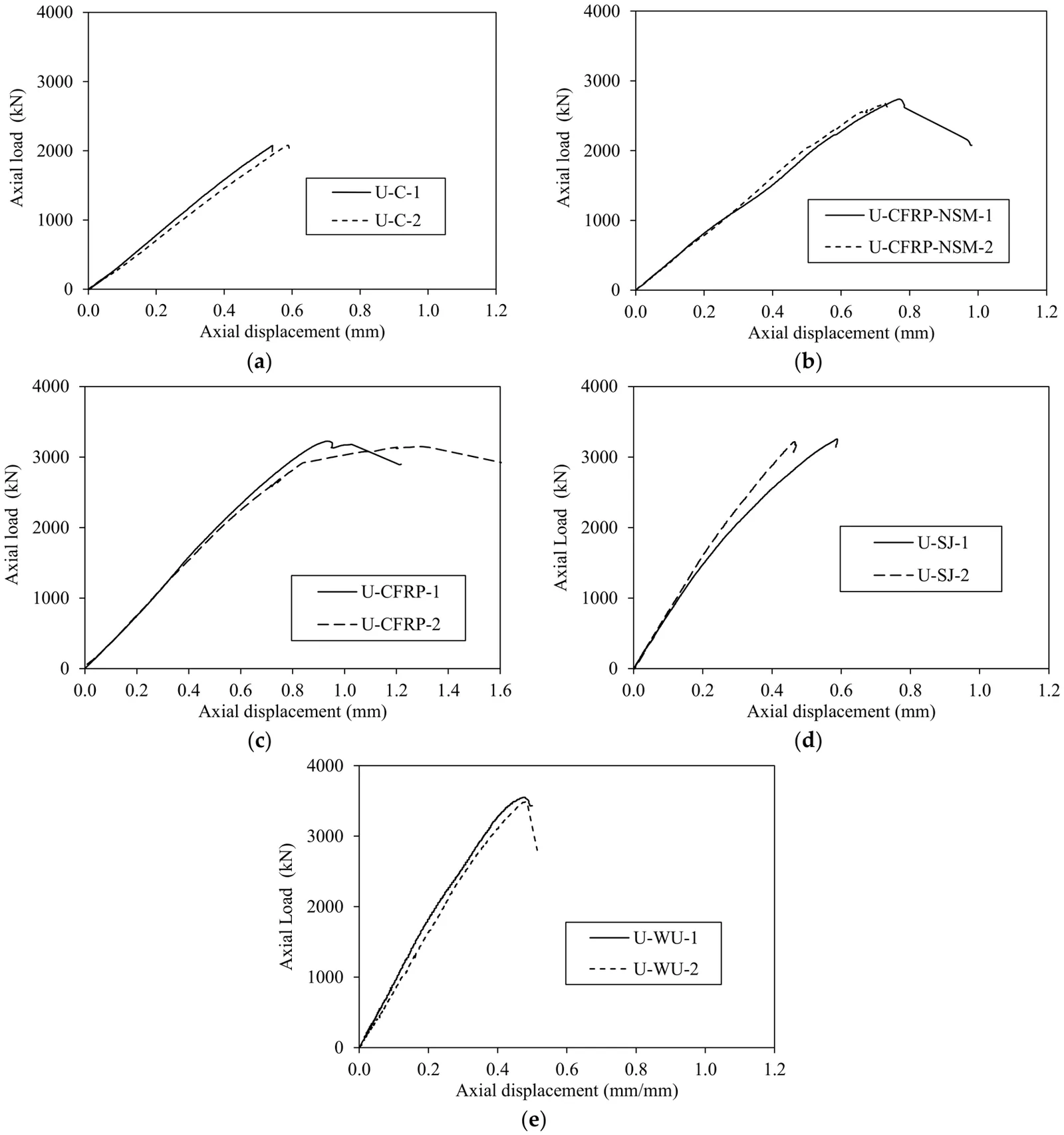
Load–displacement curves of different groups of columns: (**a**) Control U-C; (**b**) U-CFRP-NSM; (**c**) U-CFRP; (**d**) U-SJ; and (**e**) U-WU.

**Figure 9:**
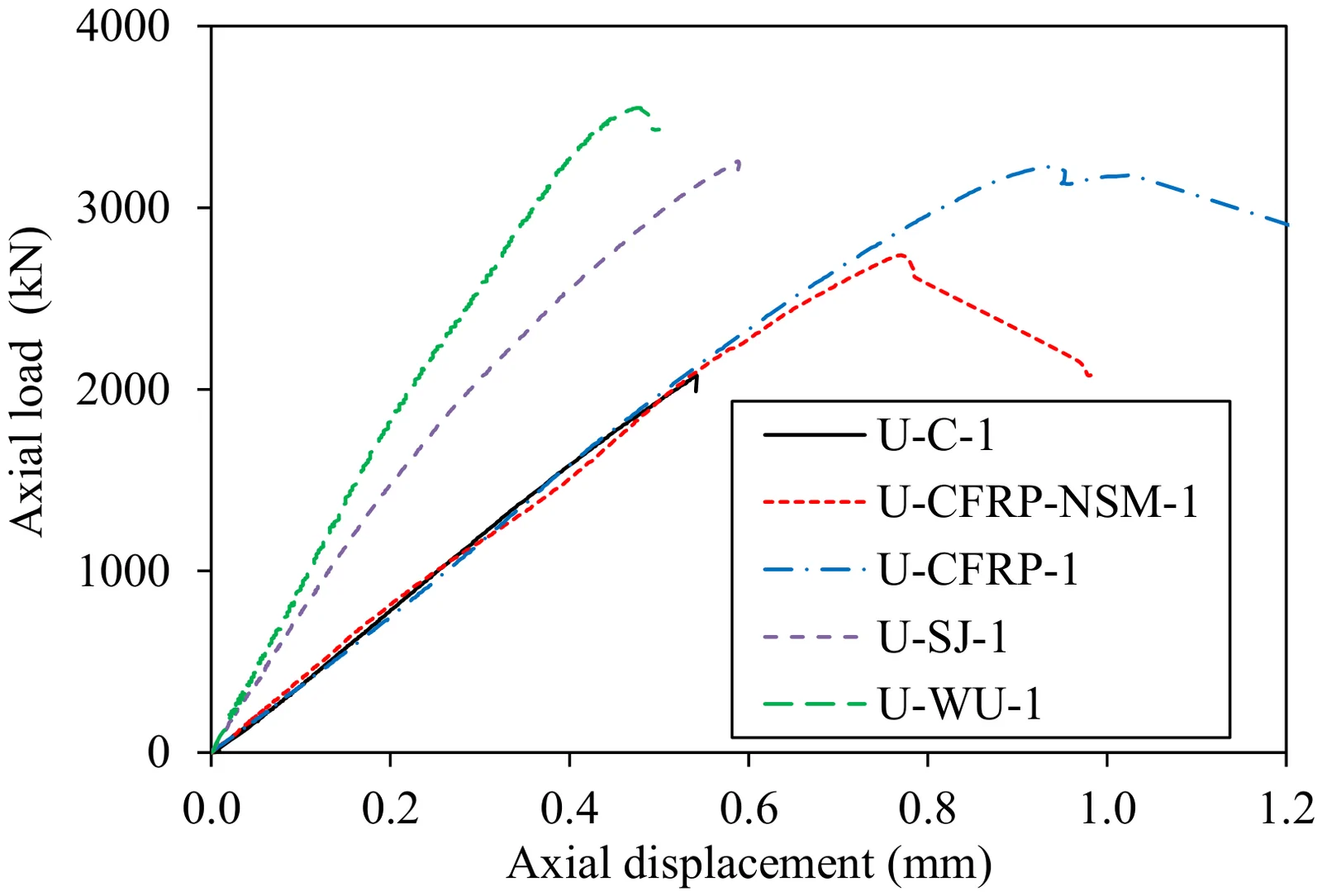
Load–displacement curves of one specimen from each group of columns.

**Figure 10:**
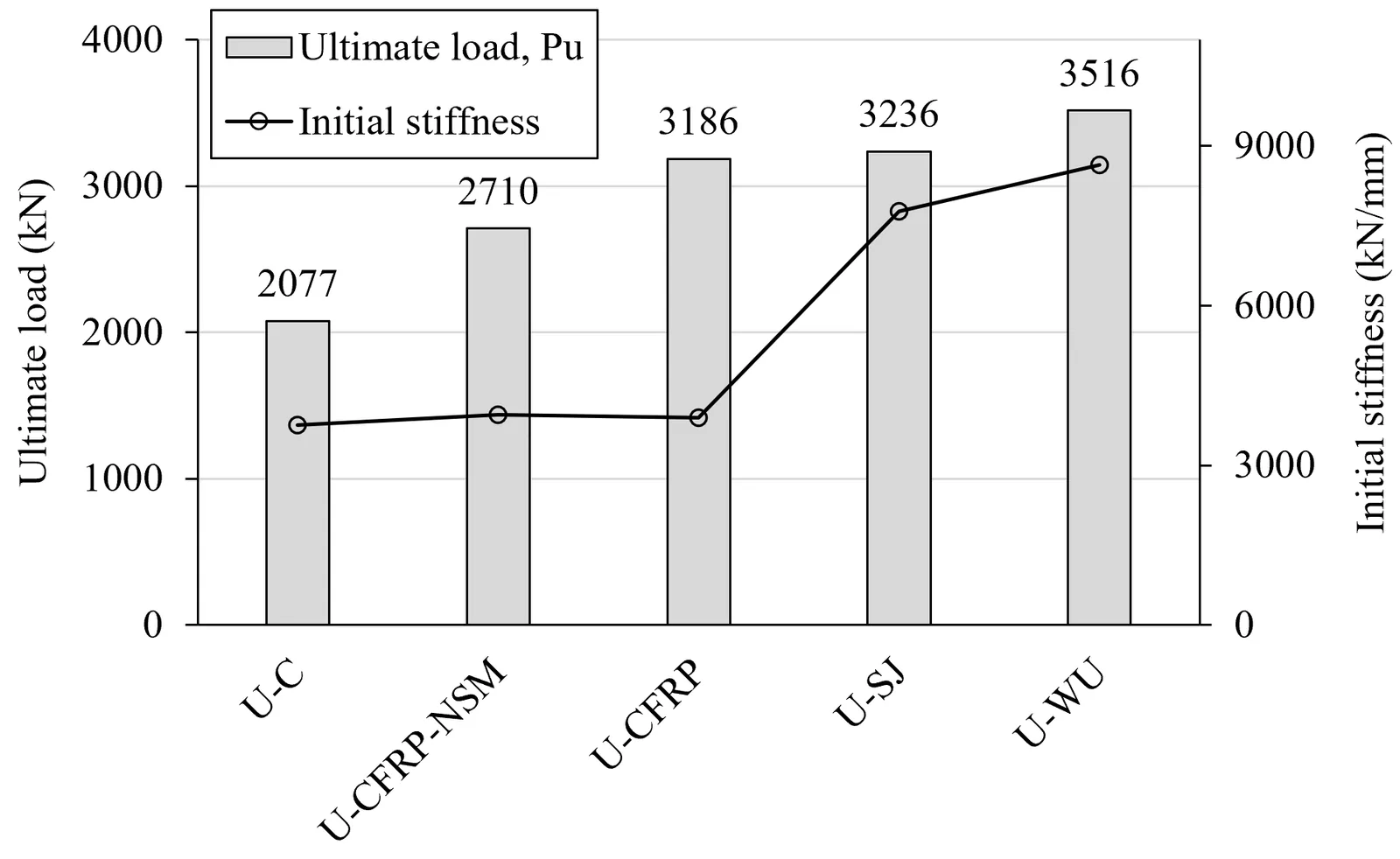
Average ultimate load and initial stiffness of all tested columns.

Figure 8 presents the axial load–compression curves for the tested columns, showing generally consistent responses between the replicate specimens. The load–compression responses of columns from different groups are compared in Figure 9. In addition, Figure 10 shows the average values of load-carrying capacities of all columns, with the corresponding values summarized in Table 3. The standard deviation (SD) and coefficient of variation (COV) are also reported in the table. The total energy absorbed was calculated as the area under the complete experimental load–compression curve. Since the strengthening schemes resulted in substantially different peak loads, the total energy was also normalized by dividing it by the corresponding peak load. This normalized energy parameter reduces the direct influence of strength and provides a more meaningful comparison of the overall deformation and post-peak response of the different strengthening schemes. The calculated average values have been added to Table 3. All specimens initially exhibited almost linear elastic behavior, where the axial displacement increased nearly proportionally to the increase in load until the specimen reached its ultimate capacity. In the post-peak region, some columns experienced a sharp and sudden drop in load with a very slight increase in displacement. However, some of the strengthened columns exhibited ductile behavior, which delayed overall failure.

Figure 9 presents the load–displacement plots of one of the specimens of each group. The control specimen (U-C) shows an almost linear response up to 2075 kN corresponding to a 0.54 mm displacement. Beyond this point, the load dropped abruptly, indicating brittle failure. The plot of the column of the G2 group (CFRP-NSM) illustrates a significant increase in the ultimate load of the U-CFRP-NSM column, reaching 2739 kN, corresponding to 0.77 mm displacement, which is 32.0% more than the U-C column. However, the failure mode was similar to the U-C column, showing a sharp drop after the ultimate load, suggesting that this scheme did not provide enough lateral support to the NSM bars to prevent sudden failure, which caused the CFRP sheets to rupture before the final failure. The curve of the U-CFRP column (G3 column) exhibited a substantial increase in load capacity. At 0.93 mm displacement, the maximum load was 3224 kN, which is 55.4% more than the U-C column. This column showed more post-peak ductility, maintaining significant load resistance as the displacement increased to approximately 1.22 mm, with a slight decrease in load (Figure 9). The three layers of wrapped CFRP sheets offered effective lateral confinement, restricting radial expansion of concrete and avoiding premature crushing. The U-SJ column (G4 column), which was retrofitted with a steel tube, had a maximum load of 3254 kN, which is 56.8% more than the U-C column. However, its post-peak response is less ductile than the CFRP-confined column (U-CFRP), as evidenced by the more rapid load drop immediately after reaching its peak at a displacement of 0.59 mm. This performance shows that the steel jacketing substantially enhanced the column strength, although deformation capacity remained limited. Among all the specimens, the U-WU column (G5 column) strengthened with UHPC jacketing reinforced with WWM and 4Ø8 longitudinal bars had the highest ultimate load capacity, reaching 3548 kN, which corresponds to a 70.9% increase over the control column (U-C), as shown in Figure 9. This strengthening scheme significantly increased strength, but it also caused a substantial decrease in displacement and ductility. The column reached its maximum load at a small displacement of 0.48 mm, after which it failed suddenly and severely. These results show that the primary contribution of this strengthening scheme was the enhancement in the effective cross-sectional area and compressive resistance. Despite its superior strength, this scheme failed to provide the necessary lateral restraint and ductility, leading to a brittle failure. The effectiveness of the strengthening schemes depends on achieving a balance between increased strength and providing effective lateral restraint to enhance ductility. Among all tested columns, the CFRP wrapping scheme (i.e., U-CFRP column) offered a balanced performance that provided significant improvements in both strength and ductility, while the strengthening scheme of the G5 column (U-WU) produced the highest strength but lacked ductility.

As far as enhancement in peak load is concerned, the investigated strengthening schemes are arranged in the order of decreasing efficiency as follows: UHPC jacket reinforced with WWM and steel rebars, steel jacket, CFRP jacket, and CFRP jacket combined with NSM steel rebars.

Table 3 shows that the replicate specimens exhibited consistent ultimate load capacities, with coefficients of variation ranging from 0.1% to 1.7%. Given the limited number of replicates (*n* = 2), these values are used only to assess repeatability within the present experimental program and should not be interpreted as a comprehensive statistical characterization of the strengthening schemes.

The total energy absorption increased from 603 kN·mm for the control specimens (U-C) to 956–3073 kN·mm for the strengthened specimens. The highest energy absorption was obtained for U-CFRP (3073 kN·mm), approximately five times that of the control, followed by U-CFRP-NSM (1331 kN·mm). After normalization by the peak load, U-CFRP and U-CFRP-NSM exhibited values of 0.96 and 0.49 mm, respectively, compared with 0.29 mm for the control. In contrast, U-SJ and U-WU showed normalized values of 0.30 and 0.29 mm, respectively, despite their higher total energy absorption. This indicates that their increase in total energy absorption was primarily associated with the enhanced load-carrying capacity, whereas CFRP confinement provided a more pronounced improvement in deformation and post-peak response.

### 3.3. Column Stiffness

The axial load resistance of columns is strongly affected not only by their ultimate load capacity but also by their initial stiffness. Increasing the initial stiffness is beneficial in columns of multi-story buildings, as it will reduce the axial shortening as well as the creep deformation. Also, it will reduce the secondary moments in long columns. Thus, the method used to strengthen columns must be capable of improving both the load-bearing capacity and initial stiffness. The stiffness is governed by the mechanical characteristics of the strengthening system, with the corresponding average values summarized in Table 4 and illustrated in Figure 10. The SD and COV values are also reported in the table. The initial stiffness corresponds to the slope of the load–displacement curve in its initial phase. It was estimated by dividing 40% of the ultimate load by the corresponding axial displacement [51].

The strengthening schemes adopted for the G2 and G3 columns did not produce a noticeable change in the initial stiffness. The U-CFRP-NSM and U-CFRP columns exhibited values comparable to the control column (U-C). This behavior may be due to the relatively thin CFRP layers, which do not significantly increase the effective cross-sectional area or alter the behavior of the uncracked concrete in the initial loading stages, as the CFRP becomes effective at higher loads. Consequently, the initial stiffness of U-CFRP-NSM and U-CFRP columns remained dominated by the mechanical properties of the unconfined concrete. In contrast, the G4 and G5 columns had significant enhancement in stiffness, with U-SJ and U-WU columns exhibiting increases of 107% and 130%, respectively, compared to the control column (U-C). As seen in Figure 10 and Table 4, the U-WU column attained the highest initial stiffness. This is mainly due to the UHPC jacketing system, which surrounds the original sample with a thick layer of material that has a high modulus of elasticity, causing a significant increase in effective cross-sectional area. Although the steel tube jacket employed in the G4 column did not add the same equivalent thickness as the UHPC jacket used for the G4 column, the U-SJ column still showed high stiffness, confirming the efficacy of steel confinement in increasing the elastic response of concrete columns.

### 3.4. Load-Strain Curves

**Figure 11:**
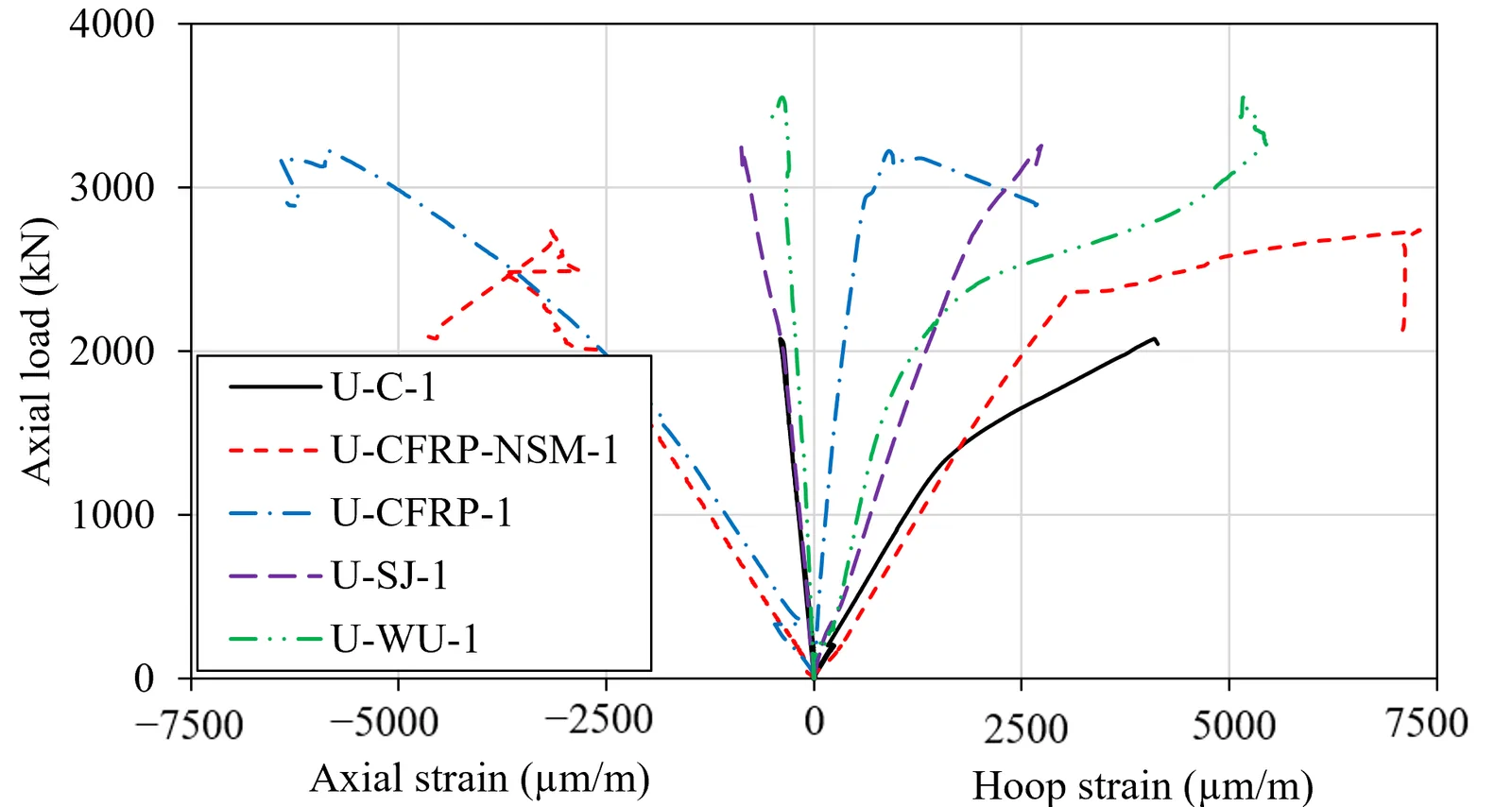
Load-strain curves of all tested columns.

Figure 11 presents the measurements obtained from the strain gauges for one of the columns of each group. The figure illustrates the variation in strains with the axial load. The strain data were extracted from transverse and axial strain gauges placed on the concrete surface and various confinement materials, including steel tube, CFRP, and UHPC jackets. During the test, observations indicated that the formation of cracks, especially the larger ones, led to significant deformations and concrete damage within the specimen cross-section, which were adjacent to the gauges in some of the specimens. This affected some strain gauge readings.

As axial compression increased, the concrete core cracking caused radial expansion, exerting lateral pressure on the confinement material. Consequently, the confinement materials experienced biaxial strain due to the axial load and hoop strains generated by the concrete exerting outward pressure on the jacket. In contrast, the concrete was subjected to triaxial stress as a result of the restraining action of the surrounding confinement jacket, which significantly influenced its deformation and failure response.

During the initial loading phase, all observed strains showed an almost linear increase, reflecting the elastic behavior of the specimens. Within this stage, the transverse strains of the confinement jackets were relatively small. As axial load increased, the strains in the external confinement materials gradually approached their yield limits, and the slope of the load-strain curves decreased until the loads attained their maximum threshold, as shown in Figure 11. The findings indicate that the confinement materials effectively supported the core concrete and increased the load capacity of columns. Figure 11 shows that the U-CFRP specimens had the highest strain levels in comparison with all other columns. This finding indicates that strengthening the specimens using CFRP provided more confinement prior to failure compared with the other schemes. The findings demonstrated that U-CFRP specimens exhibited high ductility and energy absorption, which is confirmed by the load–displacement curves (refer to Figure 9), where these specimens attained the largest displacements before failure.

The specimens investigated in this study were reduced-scale short columns; therefore, the reported strength enhancements should not be directly extrapolated to full-scale structural columns. Increasing the member size may affect CFRP confinement efficiency, bond and development of NSM reinforcement, and stress transfer between the existing column and the external jacket. Moreover, slenderness and second-order effects, which may become important in full-scale columns, were outside the scope of the present investigation. Accordingly, the results primarily provide a comparative assessment of the investigated strengthening schemes at the tested scale, and further large-scale testing is required to establish their quantitative applicability to full-scale structural members.

## 4. Analytical Modeling

In this research, the peak load of control and strengthened columns was computed using the equations of both ACI 318-25 [52] and ACI 440.2R-17 guidelines [53]. The calculation procedures adopted for the different column groups are described in the following subsections. The selected formulations provide a consistent and practical approach for evaluating the tested strengthening configurations. For FRP-confined columns, the formulation adopted from ACI 440.2R-17 [53] is based on established FRP confinement models, including the Lam and Teng model [54], and therefore incorporates established principles governing the confinement behavior of FRP-wrapped concrete. For steel-jacketed columns, the well-established confinement model of Mander et al. [55] was used. Alternative confinement formulations may produce somewhat different predictions because of differences in confinement effectiveness and constitutive assumptions; however, a comprehensive comparison of alternative models is not in the scope of this study.

### 4.1. Group G1

For unstrengthened columns in group G1 (U-C), the ACI 318-25 code [52] was employed to calculate the axial capacity using:

(1) $$P_{u}=0.85f_{c}^{\prime }\left(A_{g}-A_{\mathrm{st}}\right)+A_{\mathrm{st}}f_{y,\mathrm{st}}$$

where *P_u_* is the maximum load; $f_{c}^{\prime }$ is the specified compressive strength of concrete; *A_g_* and *A_st_* are, respectively, the area of the column section and main steel bars; and *f_y_*_,_*_st_* is the yield strength of longitudinal steel bars.

### 4.2. Groups G2 and G3

For strengthened columns in group G2 (U-CFRP-NSM) and group G3 (U-CFRP), the first step is to assess the confined compressive strength of concrete ($f_{\mathrm{cc}}^{\prime }$) using the following equations of the ACI 440.2R-17 guidelines [53].

(2) $$f_{\mathrm{cc}}^{\prime }=f_{c}^{\prime }+3.3f_{c}^{\prime }\Psi_{f}k_{a}f_{l}$$

where Ψ*_f_* = FRP strength reduction factor (=0.95); and *k_a_* = efficiency factor for FRP confinement = 1.0 for circular columns, and $f_{l}$ is the maximum confinement pressure provided by the FRP jacket, which is given by:

(3) $$f_{l}=\frac{2E_{f}n_{f}t_{f}{ℇ}_{\mathrm{fe}}}{D}$$

where *E_f_* = tensile modulus of elasticity of FRP material; *n_f_* = number of FRP wrapping layers; *t_f_* = thickness of one layer of CFRP; ℇ*_fe_* = effective strain of FRP material (taken as 0.55ℇ*_fu_*, where ℇ*_fu_* is the rupture strain of FRP); and *D* = column diameter. For strengthened columns of group G2, the peak axial capacity is estimated from

(4) $$P_{u}=0.85f_{\mathrm{cc}}^{\prime }\left(A_{g}-A_{\mathrm{st}}-A_{\mathrm{st},\mathrm{NSM}}\right)+A_{\mathrm{st}}f_{y,\mathrm{st}}+A_{\mathrm{st},\mathrm{NSM}}f_{y,\mathrm{NSM}}$$

where *A_st_*_,_*_NSM_* and *f_y_*_,_*_NSM_* are, respectively, the area and yield strength of steel NSM rebars. For CFRP-upgraded columns in group G3, the ultimate load is estimated from

(5) $$P_{u}=0.85f_{\mathrm{cc}}^{\prime }\left(A_{g}-A_{\mathrm{st}}\right)+A_{\mathrm{st}}f_{y,\mathrm{st}}$$

### 4.3. Group G4

For steel-jacketed columns in group G4 (U-SJ), the first step is to compute the confined compressive strength of concrete $f_{\mathrm{cc}}^{\prime }$ using the model developed by Mander et al. [55] for steel-confined concrete.

(6) $$f_{\mathrm{cc}}^{\prime }=f_{c}^{\prime }\left(2.254\sqrt{1+\frac{7.94f_{l}}{f_{c}^{\prime }}}-\frac{2f_{l}}{f_{c}^{\prime }}-1.254\right)$$

where $f_{l}$ is the maximum confinement pressure provided by the steel jacket, given by

(7) $$f_{l}=\frac{2f_{\mathrm{yj}}t_{j}}{D^{\prime }}$$

where *f_yj_* = yield strength of the steel jacket; *t_j_* = thickness of steel jacket; and *D′* = diameter of confined concrete core = *D* + 2*t_g_*, where *D* = column diameter and *t_g_* = thickness of grout (=30 mm). The ultimate load of the column is then assessed using Equation (5).

### 4.4. Group G5

For columns in group G5 (U-WU), upgraded with UHPC jacketing reinforced with 3 WWM layers and 4Ø8 longitudinal steel rebars, the first step in the analysis is to compute the equivalent thickness of the WWM layer from

(8) $$t_{\mathrm{eq},W}=\frac{A_{\mathrm{bar}}}{s}$$

where *A_bar_* = area of a single bar and *s* = grid spacing. Then, the maximum confinement stress provided by WWM is estimated from

(9) $$f_{l}=\frac{2f_{y,W}n_{W}t_{\mathrm{eq},W}}{D}$$

where *f_y_*_,_*_W_* = yield strength of WWM reinforcement and *n_W_* = number of WWM layers. The compressive strength of confined concrete is then determined using Equation (6) of Mander’s model for steel-confined concrete [55]. The ultimate load of the upgraded column is estimated by superposing the calculated contributions of the original column and the UHPC jacket, as follows:

(10) *P_u_* = *P_u_*_,_*_c_* + *P_u_*_,_*_j_*

where *P_u_*_,_*_c_* = peak load shared by the original column estimated as per Equation (5); and *P_u_*_,_*_j_* = ultimate load contributed by the jacket, assessed from

(11) $$P_{u,j}=0.85f_{c,j}^{\prime }\left(A_{c,j}-A_{\mathrm{st},j}-A_{W}\right)+A_{\mathrm{st},j}f_{y,j}$$

where *A_c_*_,_*_j_* and $f_{c,j}^{\prime }$ are, in turn, the gross area and compressive strength of jacket concrete; *A_st_*_,_*_j_* and *f_y_*_,_*_j_* are, respectively, the area and yield strength of steel rebars added in the jacket; and *A_W_* is the area of the WWM layers inside the jacket = *πn_W_t_eq_*_,_*_W_D*. This formulation assumes that the calculated capacities of the original column and the UHPC jacket can be mobilized concurrently at the ultimate load. Accordingly, Equation (10) represents a simplified capacity-based superposition approach and does not explicitly account for strain compatibility, differential deformation, interface bond/slip, or local load-transfer mechanisms between the existing column and the UHPC jacket. Recent studies on confined composite and FRP-strengthened columns [56,57] further highlight the importance of strain compatibility, confinement effectiveness, reinforcement contribution, and interaction mechanisms in the axial response of strengthened concrete members. These aspects are not explicitly incorporated into the present closed-form formulation and may contribute to differences between the predicted and measured capacities. Therefore, the G5 analytical prediction should be interpreted as a practical estimate of the ultimate axial capacity rather than as a detailed representation of the complete core–jacket interaction.

**Figure 12:**
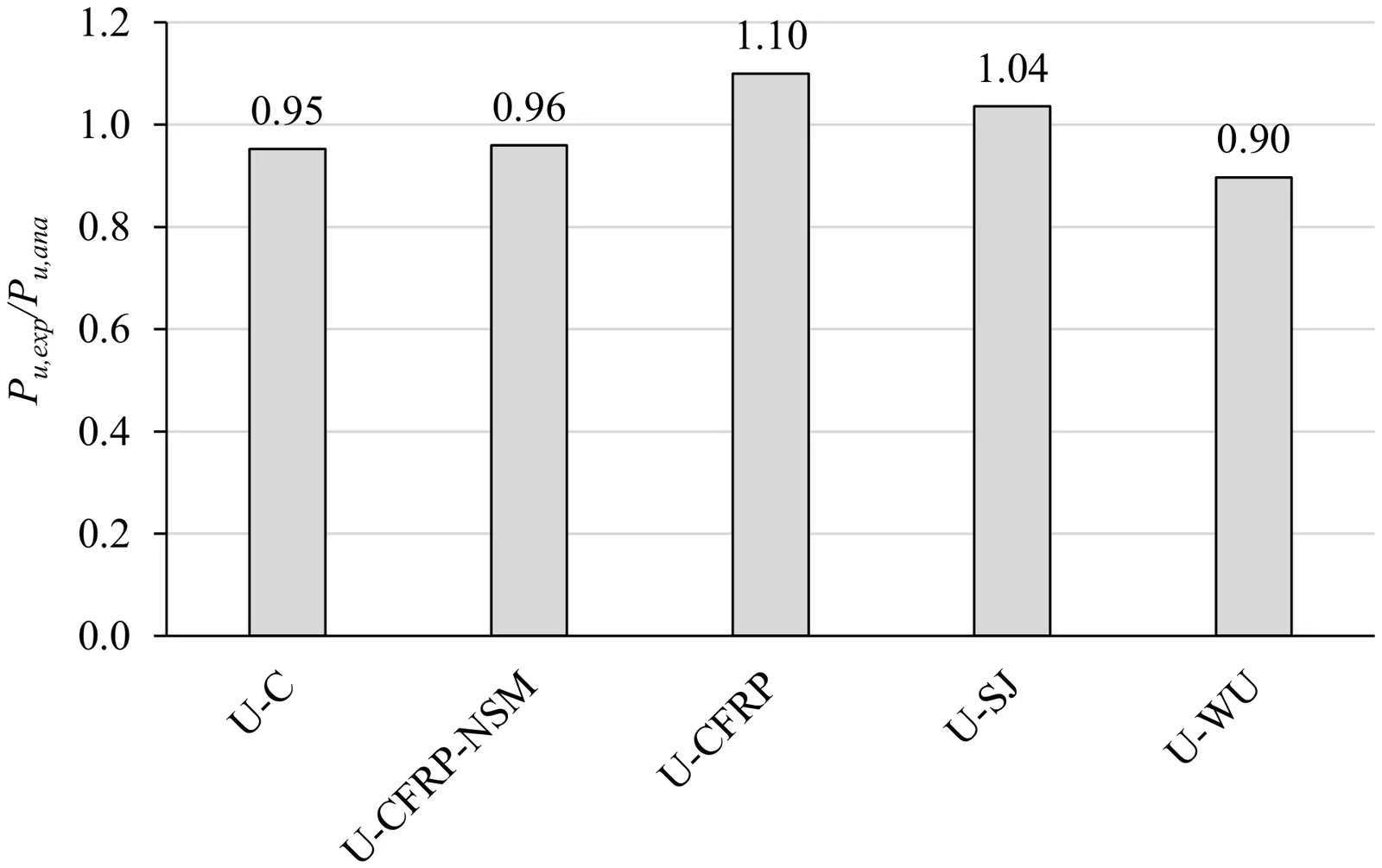
Comparison of experimental and predicted peak loads of tested columns.

Table 5 presents the peak loads predicted using the analytical procedure described above for the five column groups tested in this study, together with the corresponding test results. A comparison of the experimental-to-predicted peak-load ratios (*P_u_*_−_*_exp_*/*P_u_*_−_*_ana_*) for Groups G1–G5 is shown in Figure 12. As observed from Table 5, the proposed analytical procedure overestimated the peak load for Groups G1, G2, and G5, whereas it underestimated the peak load for Groups G3 and G4. For the unstrengthened columns of Group G1, the prediction error was approximately 5%. For the strengthened columns of Groups G2 and G4, the predicted peak loads were in close agreement with experimental results, with prediction errors of approximately 4%. For the upgraded columns of Groups G3 and G5, the prediction errors were approximately 10%. In particular, for Group G5, the analytical peak load was 3923 kN, compared with the experimental value of 3516 kN, corresponding to an overestimation of approximately 11.6% and an experimental-to-predicted load ratio of 0.90. This relatively larger overestimation may be attributed, in part, to the simplified treatment of the original-column and UHPC-jacket contributions in Equation (10), which does not explicitly capture the complex interaction and load-sharing mechanisms between the existing column and the UHPC jacket. Nevertheless, the overall comparison indicates reasonable agreement between the predictions and the experimental peak capacities of the tested columns.

**Table 5:** Prediction of peak load of tested columns using analytical models *.

Group	Strengthening Scheme	*P_u,Exp_* (Kn)	*P_u, Ana_* (Kn)	*P_u, Exp_*/*P_u, Ana_*
G1 (U-C)	Unstrengthened	2077	2182	0.95
G2 (U-CFRP-NSM)	CFRP + NSM	2710	2825	0.96
G3 (U-CFRP)	CFRP jacket	3186	2897	1.10
G4 (U-SJ)	Steel jacket	3236	3125	1.04
G5 (U-WU)	Reinforced UHPC jacket + WWF	3516	3923	0.90

* *P_u, exp_* = Average peak load from test results, and *P_u, ana_* = peak load from analytical results.

It should be noted that the present validation is based on the five group-average test results obtained in this study. Broader validation using an independent database with comparable column geometry, concrete/UHPC properties, strengthening systems, reinforcement details, and loading conditions is required to further assess the general applicability of the proposed analytical procedure and is recommended for future research.

From a practical perspective, selection of the strengthening scheme should consider both the required structural enhancement and construction constraints. CFRP wrapping (G3) provides substantial strength enhancement while adding negligible dimensions and exhibits a sustained post-peak response, making it attractive where preserving existing column dimensions is important. The combined NSM steel–CFRP scheme (G2) provides additional longitudinal reinforcement but requires groove cutting and more extensive surface preparation. Steel jacketing (G4) provides robust confinement but increases the column dimensions and structural weight and involves more demanding installation. UHPC jacketing with WWM and longitudinal reinforcement (G5) produced the greatest increase in axial capacity and stiffness but resulted in the largest increase in cross-sectional dimensions and requires more extensive construction. Thus, the preferred technique depends on whether the governing requirement is strength and stiffness enhancement, deformation capacity, minimum increase in member dimensions, or ease of installation.

## 5. Conclusions

Based on the results of the present experimental study, the following key conclusions can be drawn:

• The presence of CFRP in the U-CFRP-NSM and U-CFRP columns provided effective lateral confinement, transforming the failure mode from sudden brittle crushing to a more ductile response compared with the other strengthening techniques. CFRP wrapping improved post-peak behavior and enhanced the integrity of the column after reaching the peak load.
• The use of various strengthening schemes significantly increased the ultimate load, with the increase ranging from 30% to 69% relative to the control column (U-C).
• The G4 and G5 columns showed significant increases in initial stiffness, with U-SJ and U-WU columns exhibiting increases of 107% and 130%, respectively, compared with the control column (U-C). This indicates that strengthening techniques that enhance cross-sectional area and composite action are most effective in increasing the column’s initial stiffness.
• In terms of overall efficiency, the strengthening scheme used in G5 columns (UHPC jacketing reinforced with WWM and 4Ø8 longitudinal bars) was the most efficient in increasing both the load capacity and stiffness. However, CFRP wrapping (G3 columns) remains the optimal choice due to its superior load capacity and its ability to provide ductility without increasing the column dimensions and to maintain the integrity of the column after the peak.
• A simplified analytical approach was employed to predict the peak axial load capacities of unstrengthened and strengthened columns tested in this research. For most of the specimens, the proposed analytical models marginally overestimated the ultimate load, and the errors in prediction varied from 4% to 10%.

## Figures and Tables

**Table 1:** The properties of CFRP, GFRP sheets, and the epoxy used in this study.

Material	Characteristics	Value	Source
CFRP sheets	Tensile strength	1122 MPa	Experimental values
	Elastic modulus	68,900 MPa	
	Ultimate tensile strain	1.7%	
GFRP sheets	Tensile strength	464 MPa	Experimental values
	Elastic modulus	28,900 MPa	
	Ultimate tensile strain	1.6%	
Epoxy resin	Tensile strength	71.5 MPa	Manufacturer’s data sheet
	Ultimate tensile strain	5.25%	
	Elastic modulus	18,600 MPa	

**Table 2:** Test matrix.

Group	Specimens ID *	Strengthening Scheme	No. of Columns
G1	U-C-*i*	None (Control specimens)	2
G2	U-CFRP-NSM-*i*	4Ø12 NSM steel rebars + wrapping with 2 CFRP layers	2
G3	U-CFRP-*i*	Wrapping with 3 CFRP layers	2
G4	U-SJ-*i*	Steel jacketing with a 3 mm thick steel pipe	2
G5	U-WU-*i*	UHPC jacketing reinforced with 3 WWM layers + 4Ø8 longitudinal steel rebars	2
		Total No. of specimens =	10

* *i* = 1, 2.

**Table 3:** Summary of results (average of replicates) of column specimens.

Specimens ID	Ultimate Load		∆ At Ultimate Load (Mm)	∆_Max_ (Mm)	Total Energy Absorbed	
	(Kn) *	Deviation From U-C			(Kn.Mm)	Normalized (Mm)
U-C	2077 ± 2 (0.1%)	-	0.56	0.59	603	0.29
U-CFRP-NSM	2710 ± 41 (1.5%)	+30%	0.75	0.86	1331	0.49
U-CFRP	3186 ± 53 (1.7%)	+53%	1.11	1.42	3073	0.96
U-SJ	3236 ± 26 (0.8%)	+56%	0.53	0.53	956	0.30
U-WU	3516 ± 44 (0.3%)	+69%	0.48	0.51	1026	0.29

* Values within brackets are the COV.

**Table 4:** Initial stiffness of column specimens.

Specimens ID	Initial Stiffness	
	(Kn/Mm) *	Deviation from Control (U-C)
U-C	3760 ± 246 (6.6%)	-
U-CFRP-NSM	3949 ± 8 (0.2%)	+5%
U-CFRP	3889 ± 28 (0.7%)	+3%
U-SJ	7776 ± 417 (5.4%)	+107%
U-WU	8642 ± 726 (8.4%)	+130%

* Values within brackets are the COV.

## Data Availability

All data and models generated or used during the study appear in the article.
